# Pediatric sex estimation using AI-enabled ECG analysis: influence of pubertal development

**DOI:** 10.1038/s41746-024-01165-x

**Published:** 2024-07-02

**Authors:** Donnchadh O’Sullivan, Scott Anjewierden, Grace Greason, Itzhak Zachi Attia, Francisco Lopez-Jimenez, Paul A. Friedman, Peter Noseworthy, Jason Anderson, Anthony Kashou, Samuel J. Asirvatham, Benjamin W. Eidem, Jonathan N. Johnson, Talha Niaz, Malini Madhavan

**Affiliations:** 1 Department of Pediatric and Adolescent Medicine, Division of Pediatric Cardiology, Mayo Clinic, Rochester, MN USA; 2 Department of Cardiovascular Medicine, Mayo Clinic, Rochester, MN USA

**Keywords:** Paediatric research, Paediatrics, Paediatric research

## Abstract

AI-enabled ECGs have previously been shown to accurately predict patient sex in adults and correlate with sex hormone levels. We aimed to test the ability of AI-enabled ECGs to predict sex in the pediatric population and study the influence of pubertal development. AI-enabled ECG models were created using a convolutional neural network trained on pediatric 10-second, 12-lead ECGs. The first model was trained de novo using pediatric data. The second model used transfer learning from a previously validated adult data-derived algorithm. We analyzed the first ECG from 90,133 unique pediatric patients (aged ≤18 years) recorded between 1987–2022, and divided the cohort into training, validation, and testing datasets. Subgroup analysis was performed on prepubertal (0–7 years), peripubertal (8–14 years), and postpubertal (15–18 years) patients. The cohort was 46.7% male, with 21,678 prepubertal, 26,740 peripubertal, and 41,715 postpubertal children. The de novo pediatric model demonstrated 81% accuracy and an area under the curve (AUC) of 0.91. Model sensitivity was 0.79, specificity was 0.83, positive predicted value was 0.84, and the negative predicted value was 0.78, for the entire test cohort. The model’s discriminatory ability was highest in postpubertal (AUC = 0.98), lower in the peripubertal age group (AUC = 0.91), and poor in the prepubertal age group (AUC = 0.67). There was no significant performance difference observed between the transfer learning and de novo models. AI-enabled interpretation of ECG can estimate sex in peripubertal and postpubertal children with high accuracy.

The 12-lead electrocardiogram (ECG) is a mainstay in the toolkit for diagnosis and prognostication of cardiovascular diseases in children and adults. Artificial intelligence (AI) enabled interpretation of ECG in adults has not only demonstrated excellent performance in detecting cardiovascular diseases but also as a biomarker for physiologic parameters such as age and sex^1–3^. We have previously demonstrated that AI-enabled ECG interpretation in adults can predict sex with a high degree of accuracy, which has yet to be demonstrated in children^3^. This is a pioneer study on AI-enabled ECG interpretation of sex in a large pediatric population segregated by prepubertal, peripubertal, and postpubertal age groups.

It has been reported that males with Long QT Syndrome (LQTS) experience an increased risk of initial cardiac events during childhood that decrease after puberty^4^. These significant differences align with prior observations regarding the regression of LQTS phenotypic features, including QTc duration and cardiac events in males, post-puberty^5^. Furthermore, the QT interval duration demonstrates inherent dependencies on age and sex, with a convergence in QT duration between sexes during childhood, followed by a divergence in adulthood favoring shorter durations in males^6^.

Prior work has demonstrated that ECG features diverge between males and females during puberty, likely driven by the effects of sex hormones on cardiac ion channels and cardiac structure^7^. Given the profound impact of pubertal and sex-related differences in the prognostication of various cardiovascular diseases in addition to an individual’s physical, psychological, and social evolution, determining pubertal status is arguably as critical as defining age when characterizing adolescent participants in research studies^8^. Therefore, our study aimed to investigate the accuracy of AI-enabled ECG sex estimation models in a pediatric population.

## Methods

### Patient population

The data set included the first ECG from 90,133 unique pediatric patients (aged ≤18 years) recorded for various clinical indications between 1987–2022 at the Mayo Clinic, according to a Mayo Clinic Institutional Review Board (IRB) approved protocol, which complied with all relevant ethical regulations and included only patients who agreed to research through the research authorization process. The AI-enabled ECG models were trained on standard 10-second, 12-lead ECGs. All patients with a 12-lead ECG were included regardless of medical diagnosis. We used an 80-10-10 split to divide the cohort into training, validation, and testing datasets. The typical onset of puberty in United States has been reported between ages 8 and 13 for girls and ages 9 and 14 for boys, therefore we used the cutoff of age 8–14 years for the peripubertal time frame^9^. Subgroup analysis was performed on prepubertal (0–7 years), peripubertal (8–14 years), and postpubertal (15–18 years) patients. Model performance was also evaluated at each year of life to investigate model performance in relationship to age and pubertal development.

### AI model development

We developed two main distinct AI-ECG models for sex prediction in pediatric patients. The first model is a novel deep neural network trained specifically on the pediatric population. The second model utilized transfer learning from a previously described adult population-derived algorithm^3^. This method allowed us to leverage the knowledge gained from the adult population and adapt it to the pediatric population. We also developed a third model, trained on pediatric data, including only patients <8 years old.

All three models were implemented using a convolutional neural network (CNN) with the Keras Framework, TensorFlow backend, and Python programming language. The only data inputted for training were the raw digital 12-lead ECG signal and the associated self-reported sex of each individual. The network architectures were designed to handle the unique characteristics of ECG data, including spatial and temporal dimensions. The network operates by adjusting the weights of the convolutional filters during training to extract meaningful and relevant features in an unsupervised way. The architectures consisted of multiple layers, including convolutional layers, max-pooling layers, and batch normalization layers. These layers were organized into blocks, with each block followed by a nonlinear activation function, the rectified linear unit (ReLU). For each model, the first group of blocks was designed to extract temporal features from the ECG data. Subsequently, a spatial block was incorporated to fuse data from all leads. The extracted features from both spatial and temporal dimensions were then passed through a fully connected network to generate the final output. The output layers of both the transfer learning and novel deep neural network models had two outputs (male and female) with a SoftMax activation function, allowing the models to estimate the probability of an ECG belonging to a male or female patient. Hyperparameter tuning included batch size, learning rate, number of neurons in the fully connected layers, and convolutional layers during training to achieve the optimal model based on the validation set.

### Model evaluation and statistical methods

The transfer-learning model and the novel deep neural networks were evaluated using the holdout data set. As binary classifiers (male vs. female), the model outputs are the probabilities that the ECGs were obtained from female patients, denoted as P (convolutional CNN-predicted sex). Optimal thresholds were selected using the internal validation data set to maximize sensitivity and specificity in both models. The performance of the transfer-learning model and the novel deep neural networks were then compared to determine the most suitable approach for sex prediction in pediatric ECGs, using sensitivity, specificity, positive predicted values (PPV), negative predicted values (NPV), accuracy, F1 score, area under the curve (AUC) and area under the precision-recall curve (AUPRC).

In terms of results, the study cohort comprised 90,133 children with a mean age of 12 years and 46.7% were males. The race and ethnicity of the study population are shown in Supplementary Tables 1 and 2. The population was stratified by age into 3 groups: 0–7 years (prepubertal, *n* = 21,678), 8–14 years (peripubertal, *n* = 26,740), and 15–18 years (postpubertal, *n* = 41,715).

**Fig. 1:**
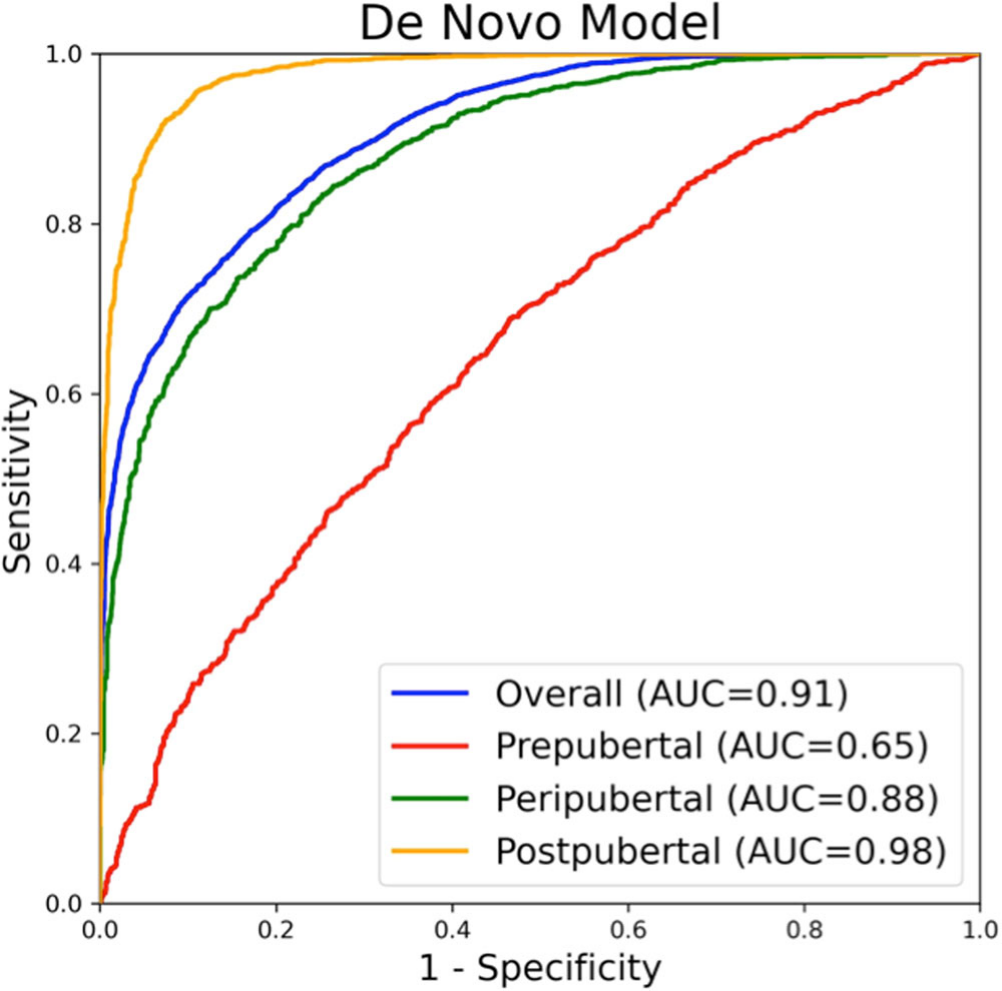
Receiver operating characteristics curves for the performance of the de novo artificial intelligence-enabled electrocardiographic model for prediction of sex in the entire cohort and stratified by age.

The two main distinct AI-enabled ECG models showed comparable performance in the entire cohort and in the age-stratified analysis. Data about the performance of the novel pediatric model is presented in Table 1 for the entire cohort and different age strata. This model demonstrated an accuracy of 81% in distinguishing female from male sex in the whole cohort with AUC of 0.91 (95% CI, 0.90–0.91) (Fig. 1). Corresponding data for the performance of the transfer-learning model are presented in Supplemental Table 3 and Supplemental Fig. 1. We further evaluated the novel pediatric model by assessing the percentage of misclassified patients broken down by age and sex, as shown in Supplementary Fig. 2.

**Table 1:** Performance of the de novo pediatric model to predict sex using artificial intelligence-enabled electrocardiography

	Overall	Prepubertal	Peripubertal	Postpubertal
AUC (95% CI)	0.91 (0.90–0.91)	0.65 (0.63–0.67)	0.88 (0.87–0.90)	0.98 (0.97–0.98)
Accuracy	0.81	0.61	0.79	0.92
Sensitivity	0.79	0.63	0.79	0.92
Specificity	0.83	0.58	0.79	0.92
Positive-predictive value	0.84	0.57	0.80	0.94
Negative-predictive value	0.78	0.65	0.78	0.90
Recall	0.79	0.63	0.79	0.92
F1 score	0.81	0.60	0.79	0.93
AUPRC	0.92	0.60	0.89	0.98

Model performance was excellent in the postpubertal age group, with an AUC of 0.98 (95% CI 0.97–0.98). Model performance was moderate in the peripubertal age group (AUC of 0.88, 95% CI 0.87–0.90) and relatively poor in prepubertal children (AUC of 0.65, 95% CI 0.63–0.67) (Table 1).

**Fig. 2:**
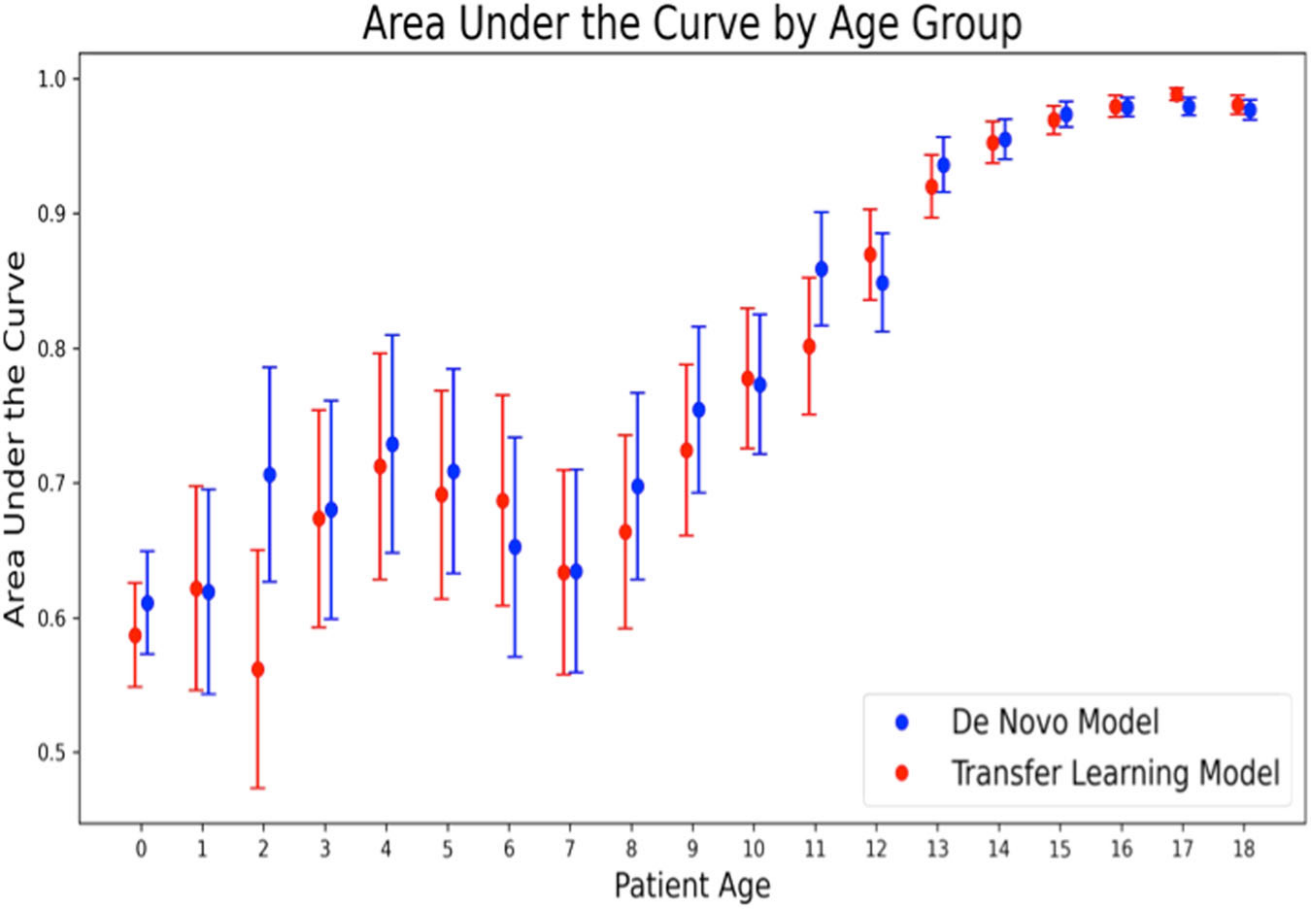
Area under the curve by age for estimation of sex using the artificial intelligence-enabled electrocardiographic de novo pediatric model (blue) and the transfer-learning model (red).

**Fig. 3:**
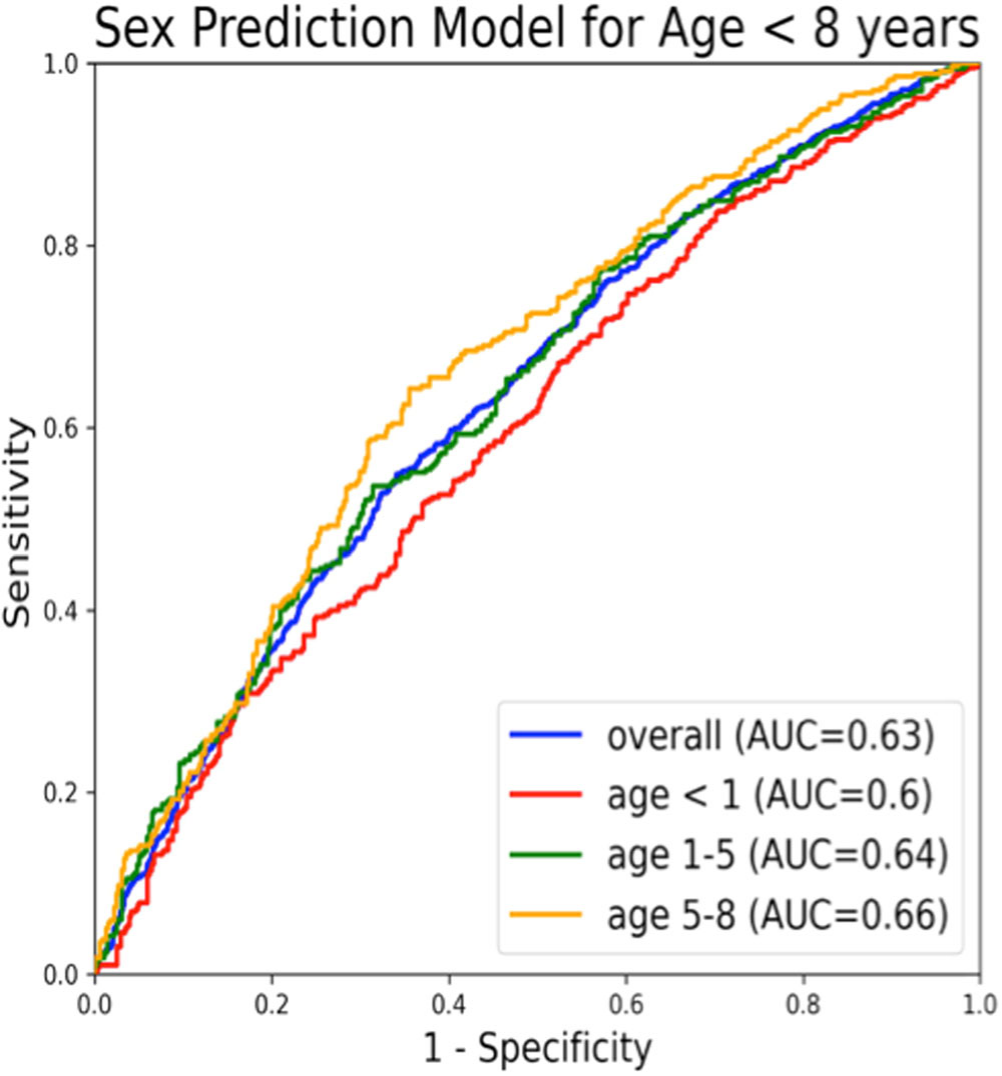
Receiver operating characteristics curves for the performance of the de novo artificial intelligence enhanced electrocardiographic model for prediction of sex in the assumed prepubertal patients, and stratified by age.

The AUC of the de novo model to estimate sex at each year of age is presented in Fig. 2. The model accuracy remains low in children below 7 years with a steep yearly increase between 7 and 14 years of age followed by a plateau in those over 15 years of age. The specific prepubertal model that was developed using only pediatric patients <8 years old, to assess whether there are unique characteristics detectable on AI-ECG in this cohort, also demonstrated poor performance, with an overall AUC of 0.63 (Fig. 3).

This study demonstrates that AI-enabled interpretation of 12-lead ECG can predict sex with good accuracy in the pediatric population. However, the algorithm performance varies by age with strong discriminatory ability in postpubertal teenagers, moderate performance in peripubertal children, and poor performance in prepubertal children. These findings demonstrate the feasibility of utilizing deep learning algorithms to detect sex-related physiologic changes in pediatric ECG.

The inability of the AI algorithm to accurately predict sex in prepubertal children not only suggests an interplay of sex hormones and their effects on various ECG parameters but also highlights a potential role of AI-enabled ECG interpretation as a non-invasive biomarker in the prediction of puberty-related changes. Prior studies have demonstrated significant sex-specific differences in measured ECG parameters like heart rate, QRS duration, and QT interval among adolescents^10,11^. While subtle differences in these parameters may be present from birth, they become significantly more apparent after puberty likely due to the influence of sex hormones on cardiac ion channel function and structure^7,12,13^. Moreover, in the adult population, AI-enabled 12-lead ECGs have not only shown the ability to identify self-reported sex with a high degree of accuracy^3^ but also demonstrated a significant correlation with total testosterone and estradiol levels^14^. In addition, male and female patients with discordance in AI-enabled ECG sex probability and self-reported sex had significantly different total testosterone and estradiol levels compared to those with concordance. Therefore, the poor performance of the AI algorithm in the prepubertal age group could be related to the lack of significant sex differences in ECG parameters among prepubertal children possibly related to the sex hormones. To address concerns regarding wide variations in ECG parameters for young children potentially leading to worse performance of our pediatric-trained AI-ECG model, we developed and trained a separate prepubertal model, which also did not show good classification discrimination between sexes.

These findings raise the possibility that with further investigation AI-enabled ECG could serve as a non-invasive biomarker for sex hormone-related pubertal effects. Further research is needed to understand the relevance of discordance between AI-predicted sex and actual sex in pediatric populations. These findings could also be leveraged as a tool for understanding or detecting certain diseases that can lead to hormonal imbalance like precocious puberty or polycystic ovarian syndrome. Similar to the adult population, there are significant sex-related differences in the presentation, progression, and outcomes of various cardiovascular diseases among children and adolescents. Puberty marks an important time frame for divulgence in the clinical outcomes of these diseases. For instance, among patients with LQTS, the risk of the first cardiac event remains higher in males until puberty whereas females remain at a high risk of the first event in adulthood^4^. With regard to Brugada syndrome, although it is autosomal dominant in inheritence, the majority of carriers who actually develop arrhythmias are adults, and the prevailing explanation is that hormonal changes taking place during puberty worsen the already unbalanced flow of ion currents that underlie Brugada syndrome^15^. Conditions such as Brugada syndrome and LQTS, with the distinct implications across different pediatric age groups, warrant re-evaluation post-puberty and potentially could be areas of research in the future in the context of AI-ECG analysis. Sex and age-related differences are also profoundly noted among children and adolescents with genetically mediated aortic diseases like Marfan syndrome in terms of aortic dimensions and risk of events^16^. As the majority of children with these disorders are followed by pediatric cardiologists, a non-invasive biomarker for sex and puberty-related changes can be a valuable tool for the management and risk stratification. From a research perspective, adolescent health and development remains mostly unaddressed and since pubertal processes have a major effect on physical, psychological, and social development, the assessment of pubertal status is at least as important as the specification of age for characterizing adolescent participants in research studies^8^.

In our study, the de novo AI-enabled ECG algorithm developed in the pediatric population had similar performance compared to utilizing transfer learning from the adult population-derived model. This suggests that transfer learning may provide a potential alternative for pediatric model development and may ease some of the sample size limitations that make the use of AI in children a challenge. Utilizing a combination of these two approaches may accelerate the adoption of this technology for children. It is important for future work to note the dramatic difference in ECGs across age groups within pediatrics and account for these changes in model development and validation. Wide variations in normal ECG parameters induced by rapid physiologic changes in very young children may create an additional challenge for training AI-enabled ECG algorithms in this population.

One key limitation of this study is our inability to confirm the pubertal status of the patients included in this study with clinical or laboratory measures of sexual development. The large numbers needed to train the CNN precluded our ability to obtain clinically documented tanner staging or concurrent sex hormone level measurements at or near the time of the ECG in this retrospective data set. A second limitation is our reliance on self-reported sex documented in the electronic medical record. This algorithm relies on a binary classification of male/female sex without having the ability to investigate a more nuanced approach including intersex individuals. While this likely affects only a small portion of the dataset, it is an area in need of further investigation. The model’s reduced accuracy in prepubertal children may reflect rapid physiological changes over these developmental years rather than a lack of distinct ECG characteristics by sex. The absence of a dedicated pediatric dataset for external validation is a limitation that we sought to mitigate by comparing the pediatric-derived model performance against a well-validated adult sex detection model, serving as a comparative framework.

In conclusion, AI-enabled interpretation of the 12-lead ECG can accurately estimate sex in peripubertal and postpubertal children, but not in prepubertal children. We showed the robustness of AI-ECG in sex discrimination in the postpubertal group, having trained and validated on a heterogeneous pediatric population. Further research is required to assess the value of AI-estimated ECG sex in the detection of pediatric cardiovascular disease, how AI-ECG can be implemented to better assess pubertal status for patients with hormonal pathologies and inclusion in future pediatric research analysis focused on sex-related risk stratification.

### Reporting summary

Further information on research design is available in the Nature Research Reporting Summary linked to this article.

### Supplementary information

Supplementary Information

Reporting Summary

## Data Availability

All requests for raw and analyzed data and related materials, excluding programming code, will be reviewed by the Mayo Clinic legal department and Mayo Clinic Ventures to verify whether the request is subject to any intellectual property or confidentiality obligations. Requests for patient-related data not included in the paper will not be considered. Any data and materials that can be shared will be released via a Material Transfer Agreement.

## Code availability

Programming code related to the data preprocessing and Keras model specification will be made available under the GNU General Public License version 3 upon request to Z.I.A. (attia.itzhak@mayo.edu).
