# Supplementary material for: Pediatric sex estimation using AI-enabled ECG analysis: influence of pubertal development
Source: NPJ Digit Med. 2024 Jul 2;7:176. doi: 10.1038/s41746-024-01165-x (PMC11220019; doi:10.1038/s41746-024-01165-x)
Supplement: Supplementary file 1 — Supplementary Information [file 41746_2024_1165_MOESM1_ESM.pdf]

Supplemental Material

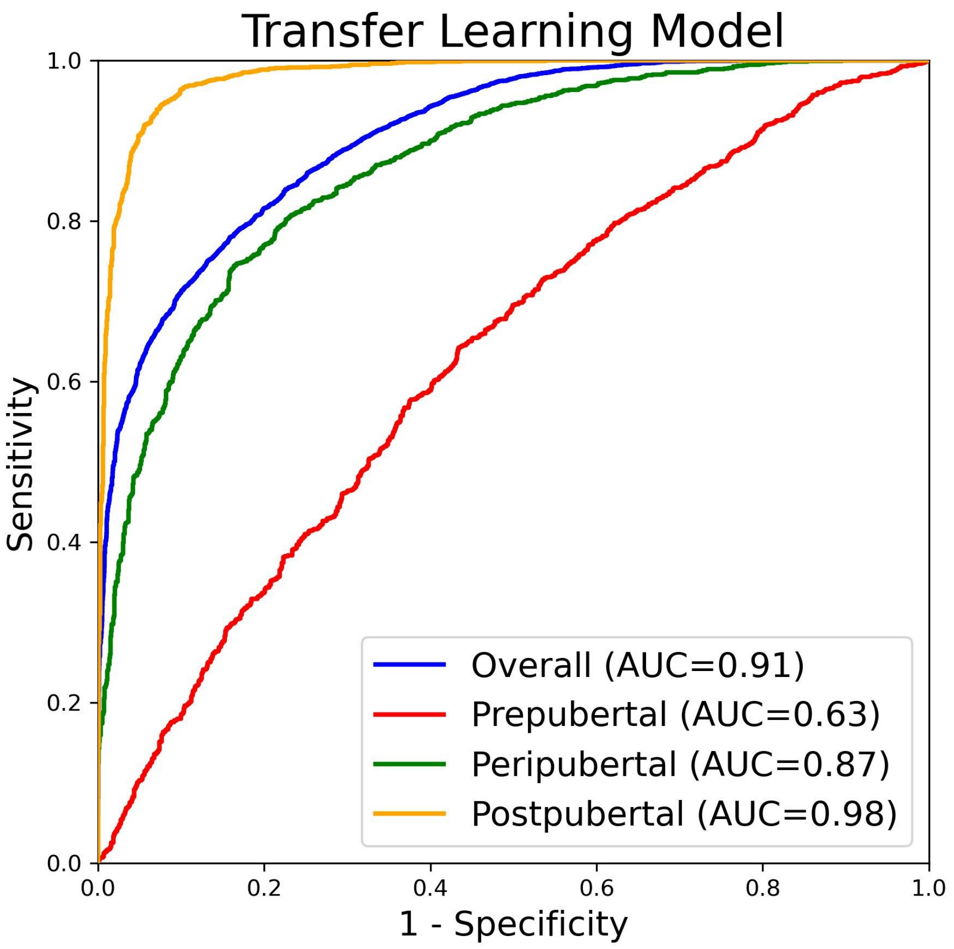

Supplemental Figure 1: Receiver operating characteristics curves for the performance of the transfer learning enabled electrocardiographic model for prediction of sex in the entire cohort and stratified by age.

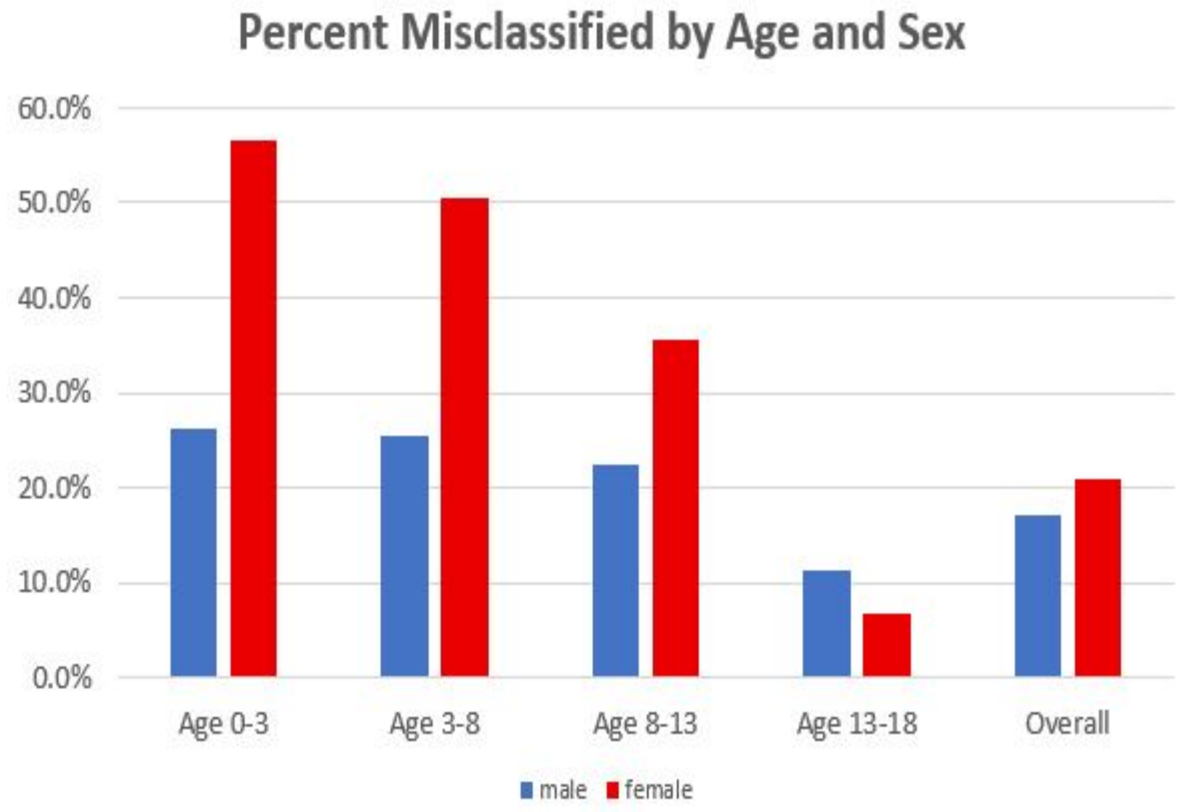

Supplementary Figure 2: Bar chart showing the frequency of misclassified patients per age grouping for the de novo pediatric model.

Supplemental Table 1: Racial distribution for pediatric study population

Race	Count	Percentage
**Caucasian**	70006	77.7
**Black**	3738	4.1
**American Indian/Alaskan Native**	607	0.7
**Native Hawaii/Pacific Islander**	124	0.1
**Asian**	1734	1.9
**Other**	3100	3.4
**Unknown**	9956	11.0
**Choose not to disclose**	715	0.8
**Unable to Provide**	95	0.1
**No Value**	58	0.1

Supplemental Table 2: Ethnic distribution for pediatric study population

**Ethnicity**	**Count**	**Percentage**
**Hispanic or Latino**	3510	3.9
**Not Hispanic or Latino**	65845	73.1
**South American**	138	0.2
**Puerto Rican**	138	0.2
**Central American**	175	0.2
**Cuban**	53	0.1
**Mexican**	660	0.7
**Other Spanish Culture of Origin Regardless of Race**	113	0.1
**Choose not to disclose**	818	0.9
**Unable to Provide**	110	0.1
**Unknown**	18511	20.5
**No Value**	62	0.1

Supplemental Table 3: Performance of the transfer-learning model to predict sex using artificial intelligence enabled electrocardiography

	Overall	Prepubertal	Peripubertal	Postpubertal
**AUC (95% CI)**	0.91 (0.90 - 0.91)	0.63 (0.61 – 0.65)	0.87 (0.86 – 0.88)	0.98 (0.98 – 0.98)
**Accuracy**	0.81	0.60	0.79	0.93
**Sensitivity**	0.79	0.64	0.79	0.94
**Specificity**	0.83	0.57	0.78	0.93
**Positive predictive value**	0.84	0.56	0.79	0.95
**Negative predictive value**	0.78	0.64	0.78	0.92
**Recall**	0.79	0.64	0.79	0.94
**F1 score**	0.81	0.60	0.79	0.94
**AUPRC**	0.92	0.57	0.88	0.98
